# Local bone quality measure and construct failure prediction: a biomechanical study on distal femur fractures

**DOI:** 10.1007/s00402-021-03782-7

**Published:** 2021-02-15

**Authors:** Dominic Gehweiler, Ursula Styger, Boyko Gueorguiev, Christian Colcuc, Thomas Vordemvenne, Dirk Wähnert

**Affiliations:** 1 AO Research Institute Davos, Clavadelerstraße 8, 7270 Davos, Switzerland; 2 Department of Trauma and Orthopedic Surgery, Protestant Hospital of Bethel Foundation, University Hospital OWL of Bielefeld University, Campus Bielefeld Bethel, Burgsteig 13, 33617 Bielefeld, Germany

**Keywords:** Distal femur fracture, Osteoporosis, Biomechanics, Local bone quality, DensiProbe

## Abstract

**Introduction:**

The aim of this investigation was to better understand the differences in local bone quality at the distal femur and their correlation with biomechanical construct failure, with the intention to identify regions of importance to optimize implant anchorage.

**Materials and methods:**

Seven fresh–frozen female femurs underwent high-resolution peripheral quantitative computed tomography (HR-pQCT) to determine bone mineral density (BMD) within three different regions of interest (distal, intermedium, and proximal) at the distal femur. In addition, local bone quality was assessed by measuring the peak torque necessary to break out the trabecular bone along each separate hole of a locking compression plate (LCP) during its instrumentation. Finally, biomechanical testing was performed using cyclic axial loading until failure in an AO/OTA 33 A3 fracture model.

**Results:**

Local BMD was highest in the distal region. This was confirmed by the measurement of local bone quality using DensiProbe^™^. The most distal holes represented locations with the highest breakaway torque resistance, with the holes on the posterior side of the plate indicating higher values than those on its anterior side. We demonstrated strong correlation between the cycles to failure and local bone strength (measured with DensiProbe^™^) in the most distal posterior screw hole, having the highest peak torque.

**Conclusion:**

The local bone quality at the distal femur indicates that in plated distal femur fractures the distal posterior screw holes seem to be the key ones and should be occupied. Measurement of the local bone strength with DensiProbe^™^ is one possibility to determine the risk of construct failure, therefore, thresholds need to be defined.

## Introduction

Fractures at the distal femur are relatively rare, representing only 6% of all femoral fractures, and they can be complicated in several situations including presence of osteoporosis or presence of a total knee endoprosthesis [1]. Approximately 50% of the distal femur fractures occur in elderly patients, with a rising number of osteoporotic and periprosthetic or peri-implant fractures observed; these represent an unresolved and growing problem in orthopedic and trauma surgery [1]. In terms of fracture morphology in the elderly, supracondylar fractures are most common due to the reduced bone quality in the metaphyseal part of the distal femur, where sufficient implant anchorage is hard to achieve [2]. Osteosynthesis of such fractures is challenging because of its higher probability to fail, especially in case of severe osteoporosis and comminuted fractures with absent medial bone contact [3, 4]. Indeed, Vallier et al. found complication rates of up to 35% in distal femur fractures treated with locking compression plates. In this cohort, the mean age of patients with complications was 64 years and all of them who developed a malunion were over the age of 55 years; presumably, a low-energy mechanism was the cause for fractures in these patients [5]. Another study by Hou et al. further demonstrated the clinical importance of this topic by reporting high complication rates of 18% in non- or malunions after locked plating and 17% following retrograde intramedullary nailing after periprosthetic distal femur fractures [6].

Several options to increase implant anchorage in the metaphyseal part of the distal femur were investigated in our group. One focus was the so-called implant augmentation, where bone cement is used to encase the screw tips and enlarge the weight-bearing surface thereby increasing the stability of the construct [7, 8]. Another approach was the investigation of alternative designs for distal locked plating. Therefore, a feasibility study was conducted, comparing a helical blade to locking screws, both with and without bone cement augmentation [9].

The primary aim of the current investigation was to better understand the variations in the local bone quality at the distal femur. Therefore, the bone mineral density (BMD) determined from high-resolution peripheral quantitative computed tomography (HR-pQCT) scans as a radiological parameter was compared to the peak torque necessary to break local trabecular structure (measured with DensiProbe^™^) as a mechanical parameter. In addition, their relation to the corresponding biomechanical plate fixation failure was investigated. A secondary aim was to identify regions of importance for enhanced implant anchorage.

## Materials and methods

### Specimens

Seven non-paired fresh–frozen human cadaveric distal femurs from female donors (4 right, 3 left) were used in this study. The mean age of the donors was 87 years (range 81–92 years).

### Local bone quality (HR-pQCT, DensiProbe^™^)

**Fig. 1:**
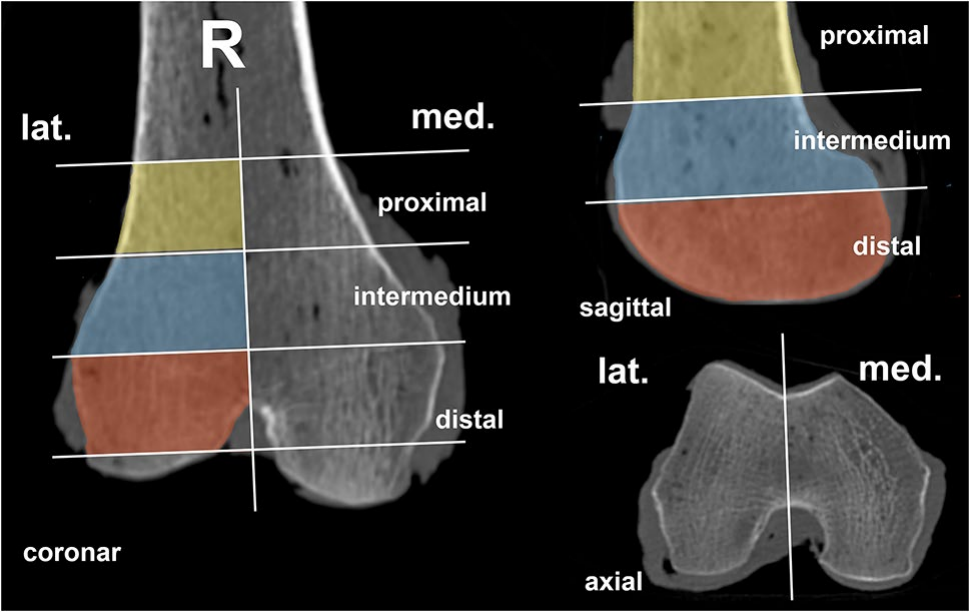
Slices of a CT scan of a right femur in the coronal, sagittal, and axial planes. The six different regions of interest (ROI) for local BMD evaluation via HR-pQCT are marked in the different planes

BMD was determined by means of HR-pQCT (XtremeCT, Scanco Medical AG, Bassersdorf, Switzerland) operated at 60 kVp, 900 µA, 750 projections, 200 ms acquisition time and at a resolution of 123 µm. BMD was evaluated for the cancellous bone in 6 regions of interest (ROI, 3 medial and 3 lateral—distal, intermedium, and proximal within the condylar region), each one of a thickness of 180 slices (Fig. 1). The location of the intermedium ROI was selected based on the largest condyle diameter. A semi-automated segmentation procedure was applied. The BMD evaluation followed an established routine and was used for further investigation. [10]

The second method for assessment of the local bone quality uses the DensiProbe^™^ device. This device characterizes local bone quality mechanically, by measuring the breakout torque of cancellous bone in a specific region. [10–13] Therefore, the 19-mm-long blade tip of the DensiProbe^™^ is hammered into the bone. After attaching a torque-measuring device, the probe is turned 90° and the maximum torque is recorded. Since this device was originally designed for intraoperative bone strength determination, the diameter of the probe (3.8 mm) is smaller than the core diameter of the implant (5 mm). This ensures a firm seat of the implant despite the measurement.

**Fig. 2:**
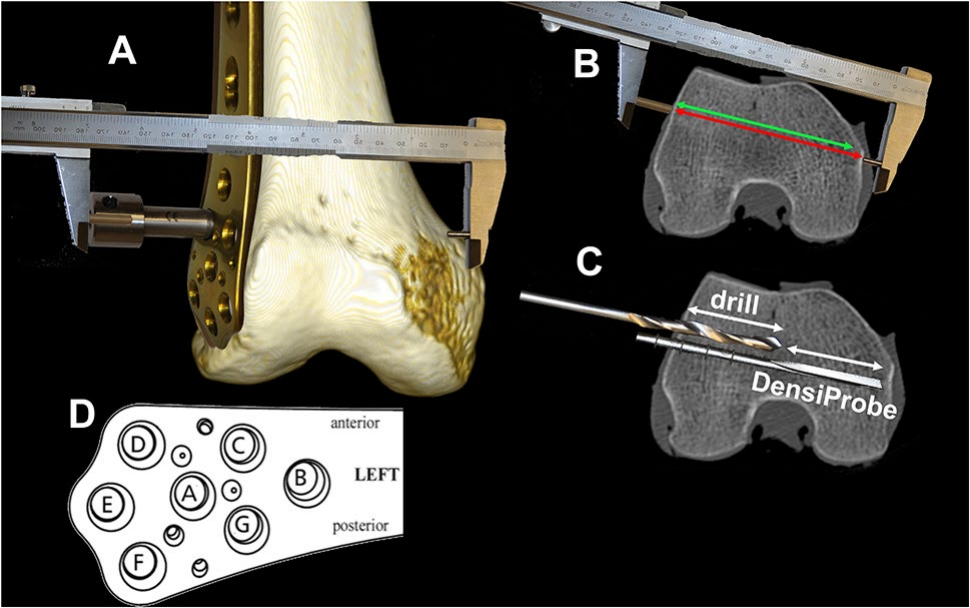
DensiProbe^™^ measurement: **a** after plate positioning and temporary fixation, the distance between both cortices is measured using a custom-made caliper. **b** The used screw length represents the next available shorter screw to the measured distance. **c** Following screw length determination, the screw hole is predrilled to a certain depth, and DensiProbe^™^ is hammered in to measure the peak torque. **d** screw hole configuration of the locking compression plate

In our study, a titanium locking compression plate for the distal femur (LCP-DF, DePuy Synthes, Zuchwil, Switzerland) was placed in the correct position and the screw holes A–G (Fig. 2d) were used to measure the breakout torque applying an algorithm adapted for distal femur application from a previous study by Röderer et al. [12]. To compare the results to the BMD within the ROIs, the plate holes have been matched as follows: hole B with medial-proximal BMD; holes A, C and G with medial-intermedium BMD; and holes D, E, and F with medial distal BMD. After temporary fixation of the plate to the bone, a modified drill sleeve was screwed into the plate hole and the distance from the plate to the medial cortex was measured using a custom-made caliper (Fig. 2a). The lengths for the drill bit and the DensiProbe^™^ device were then calculated for placement of a monocortical screw being as long as possible (Fig. 2b). The drill bit and the DensiProbe^™^ were adjusted according to these lengths, and stop rings were mounted to ensure the correct depth of insertion. The hole was predrilled in the lateral region using a 4.3-mm drill bit prior to hammering in the DensiProbe^™^ device into the medial condylar region up to the position of the measured screw tip. Using a torque-measuring device (Mecmesin Torque Sensor; Mecmesin, West Sussex, United Kingdom, accuracy of 0.032 Nm at 1 Nm) attached to probe, the peak torque in the medial region was measured by turning the latter 90° clockwise (Fig. 2c).

### Biomechanics

After removal of the DensiProbe^™^, the LCP-DF plates were fixed to the bones. Distal fixation was performed using 5 mm self-tapping locking screws (DePuy Synthes, Zuchwil, Switzerland) in all seven distal plate holes at the maximum possible length. An AO/OTA 33 A3 fracture was simulated by an osteotomy 7 cm proximal the knee joint line with a gap of 1.5 cm. The proximal part of the femur was replaced with a custom-made standardized artificial femoral shaft (diameter 30 mm; length 13 cm) made of polymethylmethacrylate (PMMA, Beracryl, Suter Kunststoffe AG, Fraubrunnen, Switzerland). Proximal plate fixation was performed in a rigid manner using three ordinary screws with nuts.

**Fig. 3:**
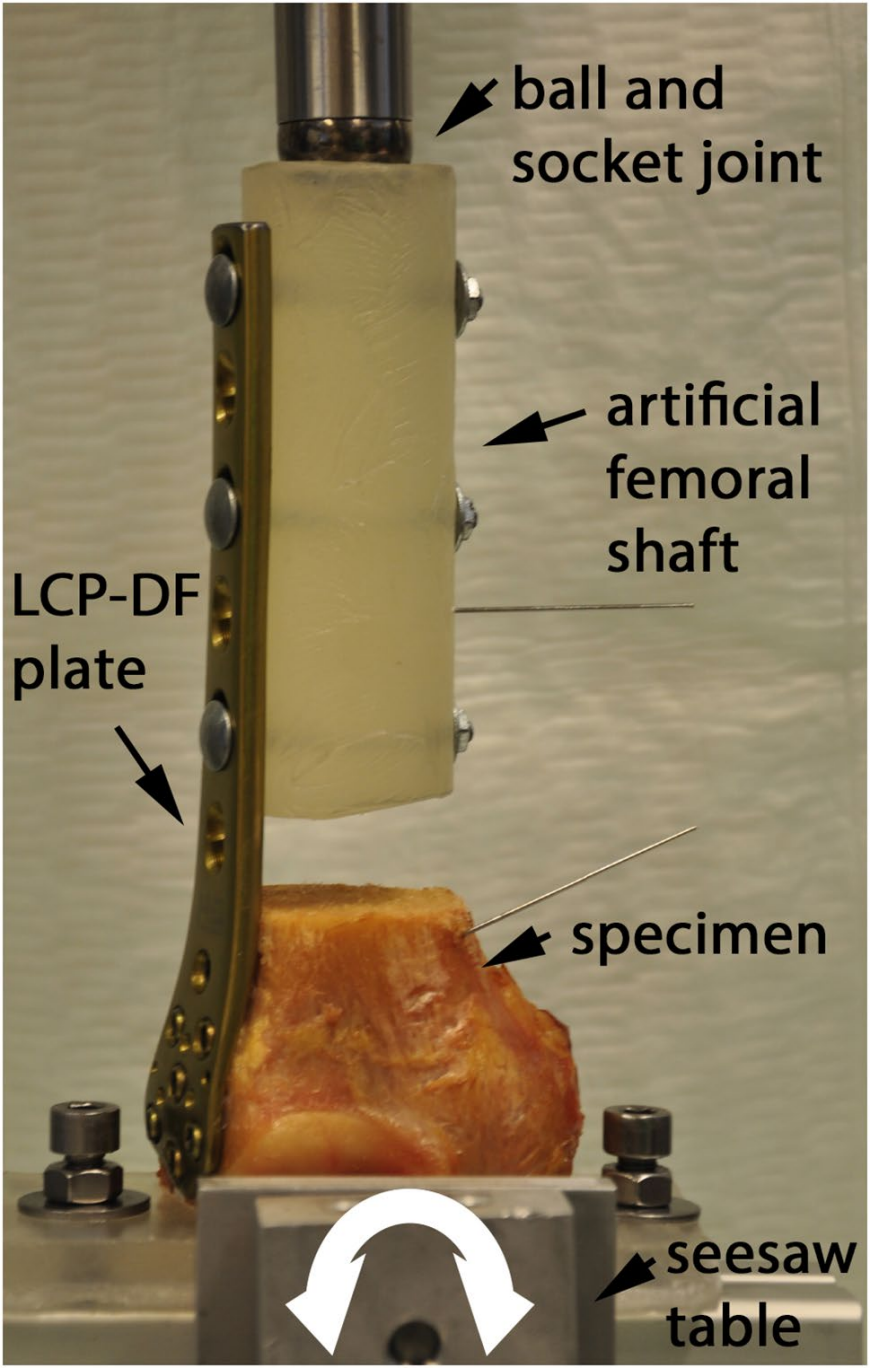
Biomechanical test setup. Specimen attached to the machine actuator via a ball-and-socket joint and placed on a seesaw table allowing medio-lateral tilting

Biomechanical testing was performed using a servo-hydraulic testing machine (MTS 858 Mini Bionix II, MTS, Eden Prairie, USA) equipped with a 4 kN load cell. The specimens were oriented vertically and attached to the machine actuator via a ball-and-socket joint proximally, whereas the distal femur part was attached to the machine base via an individual pre-shaped mold, connected to a seesaw table being able to tilt medially and laterally along the axis defined by the intercondylar notch (Fig. 3). Cyclic axial sinusoidal loading of the specimens was performed at 2 Hz until failure. Starting at a 750 N peak compression force, the load was progressively increased cycle by cycle at a rate of 0.05 N/cycle. The valley load was kept at a constant level of 100 N [7]. Number of cycles to failure was determined for each specimen based on an arbitrary failure criterion of 4° varus collapse.

### Statistical evaluation

Statistical evaluation was performed using Microsoft Excel 2016 (Version 16.16.1, Microsoft Cooperation, Redmond, USA) and SPPS software package (Version 24, SPSS, Chicago, IL, USA). Shapiro–Wilk test was applied to screen and prove normality of data distribution regarding the DensiProbe^™^, BMD and biomechanical parameters of interest. Independent *t* test was used to detect significant differences between the different bone locations with regard to each of the evaluated parameters. Pearson Correlation test was used to explore correlations among BMD, DensiProbe^™^ and biomechanical values. Significance level was set at *p* = 0.05 for all statistical tests.

### Ethical approval

The femora were obtained from a local anatomical institute and used for this examination based on the “Gesetz über das Leichen-, Bestattungs- und Friedhofswesen (Bestattungsgesetz) des Landes Schleswig–Holstein vom 04.02.2005, Abschnitt II, § 9 (Leichenöffnung, anatomisch)”, according to which it is allowed to dissect the bodies of the donors (Körperspender/in) for scientific and/or educational purposes. An additional ethical approval was not necessary.

## Results

### Bone mineral density (BMD)

**Fig. 4:**
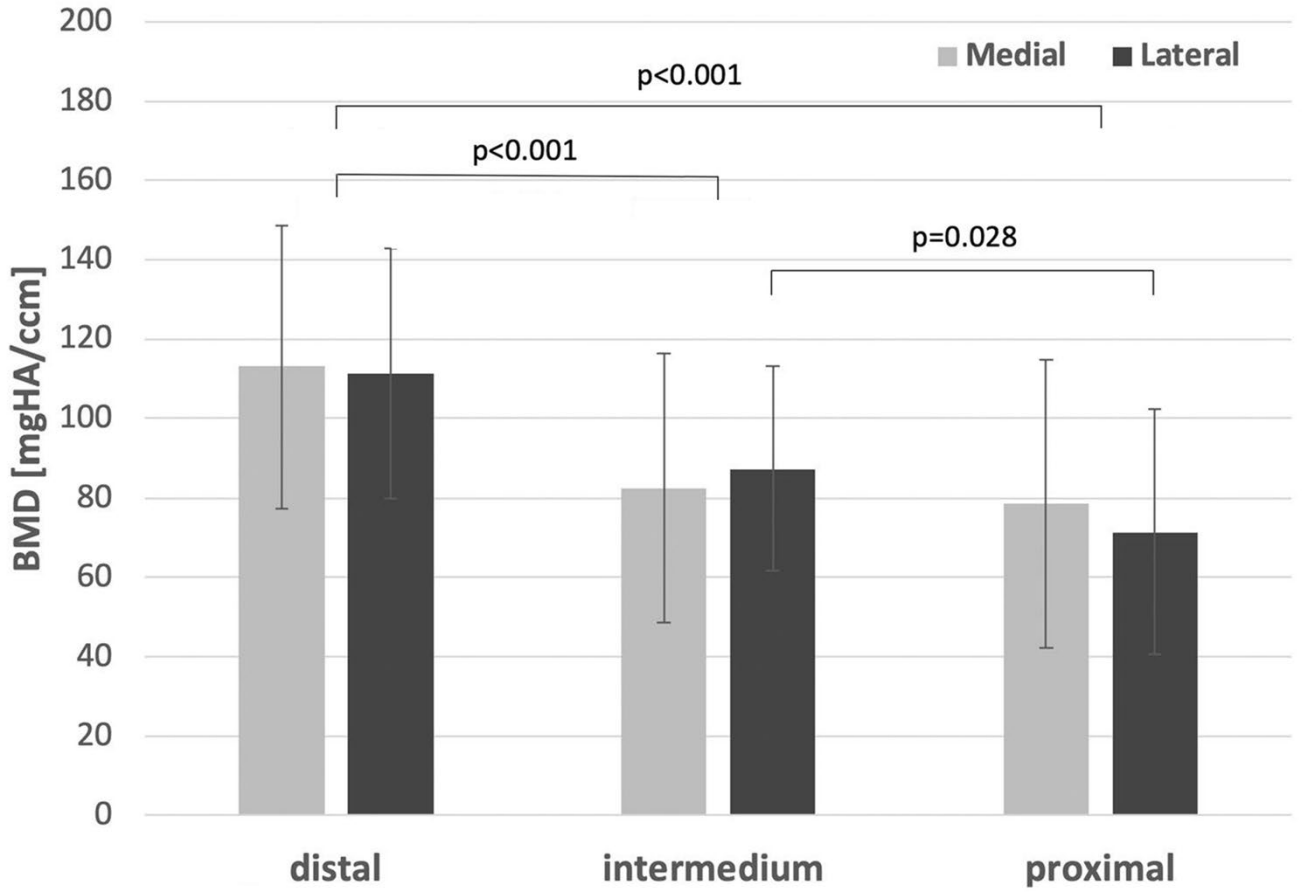
Local bone mineral density (BMD) determined from HR-pQCT scans for the different anatomical locations (regions of interest) in terms of mean value and standard deviation

Local BMD derived via HR-pQCT decreased significantly from the distal to intermedium and proximal femur in both the medial and lateral sides (*p* < 0.001, Fig. 4, Table 1). On the medial side, the intermedium and proximal regions reached only 73% and 70% of the BMD value in the distal region, while on the lateral side they reached 79% and 64%, respectively. The difference between the intermedium and proximal BMD was significantly different for the lateral side (*p* = 0.028).

**Table 1:** Bone mineral density (BMD, HR-pQCT) and peak torque (DensiProbe^™^) broken down by the region of interest as mean value and standard deviation (SD) as well as % of the maximal value

Region of interest	BMD [mgHA/ccm]		Peak torque [Nm]	
	Lateral	Medial	Medial	Plate hole
Proximal	71.5 ± 30.7 (64%)	78.6 ± 36.2 (70%)	0.22 ± 0.10 (45%)	B—0.22 ± 0.10 (34%)
Intermedium	87.4 ± 25.7 (79%)	82.4 ± 33.8 (73%)	0.37 ± 0.20 (75%)	C—0.29 ± 0.18 (45%)
				A—0.36 ± 0.15 (56%)
				G—0.48 ± 0.29 (75%)
Distal	111.3 ± 31.4 (100%)	113.0 ± 35.5 (100%)	0.50 ± 0.20 (100%)	D—0.36 ± 0.11 (56%)
				E—0.51 ± 0.17 (80%)
				F—0.64 ± 0.26 (100%)
Mean	90.1 ± 33.6	91.3 ± 38.4	0.41 ± 0.23	

### DensiProbe^™^ measurements

**Fig. 5:**
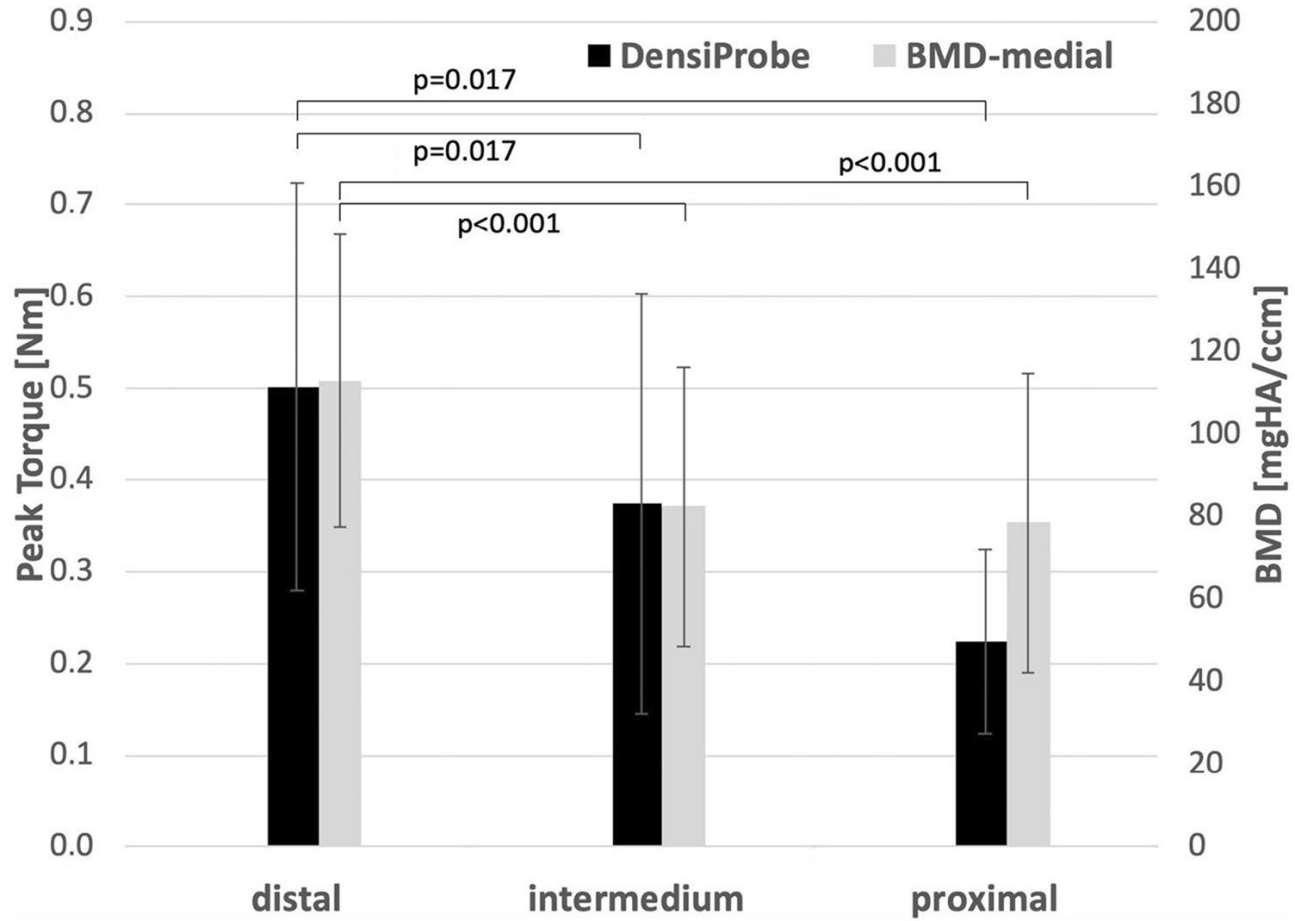
DensiProbe^™^ (peak torque) for the different regions (grouped screw holes) compared to the medial BMD (from HR-pQCT) in terms of mean value and standard deviation

Local bone strength (measured with DensiProbe^™^) corresponded well to the respective BMD measured on the medial femur side (Fig. 5, Table 1). The distal holes demonstrated the highest peak torque (*p* = 0.017), compared to the others, whereas the intermedium and proximal regions reached only 75% and 45% of the peak torque in the distal region, respectively.

Looking at the screw holes alone, independently from their matching to the BMD within the ROIs, the posterior holes (F and G) demonstrated significantly higher peak torques compared to the anterior holes (C and D; *p* = 0.001), 0.56 Nm versus 0.32 Nm on average, respectively.

When the HR-pQCT and DensiProbe^™^ data were compared, the strongest correlation was found between the mean values of both parameters for all medial ROIs from the HR-pQCT measurements considered together, and all holes together from the DensiProbe^™^ measurements (*R*^2^ = 0.863, *p* = 0.002; Table 2). In addition, the torque values from the individual hole measurements were correlated with their corresponding medial local BMD values. The strongest correlation was found for hole F (*R*^2^ = 0.793, *p* = 0.007).

**Table 2:** Correlation analysis related to the BMD and DensiProbe^™^ values, considering all medial regions of interest (proximal, intermedium, distal) and all screw holes (A, B, C, D, E, F, G)

BMD	DensiProbe^™^	*R*	*R*^2^	P-value
Mean all	Mean all	0.929	0.863	0.002
Proximal	B	0.743	0.553	0.056
Intermedium	C	0.868	0.753	0.011
	A	0.770	0.593	0.043
	G	0.675	0.455	0.096
Distal	D	0.612	0.375	0.144
	E	0.760	0.577	0.047
	F	0.891	0.793	0.007

### Biomechanics

The mean number of cycles to failure was 14,879 (standard deviation 7,091). When the number of cycles to failure was related to the local bone strength (measured with DensiProbe^™^), the strongest correlation was found for hole F (*R* = 0.84, *R*^2^ = 0.70, *p* = 0.018). With regard to BMD, number of cycles to failure demonstrated the strongest correlation with the mean medial BMD values (*R* = 0.82, *R*^2^ = 0.68, *p* = 0.023). Taking each separate anatomical region into consideration, the strongest correlation was registered with the medial distal BMD (*R* = 0.79, *R*^2^ = 0.62, *p* = 0.035).

## Discussion

Even with the latest advancements in trauma and orthopedic surgery, fractures of the distal femur remain hard to treat due to their complexity and high complication rates, especially in elderly patients. To address this problem, a better understanding of bone and fracture morphology is necessary to improve implant anchorage.

In this study, we focused on the local bone quality at the distal femur from radiologic and mechanical perspectives and their correlation with construct failure. Local BMD (measured by HR-pQCT) was highest in the distal region, whereas the proximal region reached 70% (medial) and 64% (Lateral) of the maximum. This result was confirmed by the measurement of local bone quality using DensiProbe^™^, the latter indicating 45% torque resistance along the proximal plate hole compared to the mean of the distal holes. The most distal holes revealed the highest torque resistance, with the holes on the posterior side of the plate indicating higher values than those on its anterior side. Local bone quality has an important influence on construct failure. In this investigation, we demonstrated strong correlation between the cycles to failure and local bone strength (DensiProbe^™^) in the most distal posterior screw hole (F), indicated with the highest peak torque. This screw seemed to have a predictive value on the construct performance. Compared to low bone quality, high bone quality in this region will lead to later construct failure. This result demonstrates that a feasible and applicable method for intraoperative bone quality assessment exists, but thresholds need to be defined. In contrast, the HR-pQCT measurements revealed weaker correlation between BMD and number of cycles to construct failure during biomechanical testing.

In previous work, the measurement of local bone strength using DensiProbe^™^ has been performed in vitro in various anatomical regions, including the hip, hindfoot, spine, and humerus [10, 12–16]. In agreement with our results, these studies demonstrated a good correlation between local bone strength and radiologically determined bone quality (by DEXA or HR-pQCT). In addition, similar previous biomechanical studies reported strong correlation between the local bone strength and failure load [10, 12]. Workflows for assessment of local bone strength in clinical applications during spinal and dynamic hip screw implantations have been published [17, 18]. However, techniques related to intraoperative bone quality assessment need to be further investigated and their thresholds defined. Up to now, no objective method has existed to inform the surgeon in the operating theater about the status of local bone quality. Special techniques, such as implant augmentation with bone cement, have been applied based on the subjective decision of a single surgeon; these techniques are related to additional risks for the patient and higher costs for the society.

More detailed knowledge of local bone characteristics, as well as their changes resulting from osteoporosis diseases, is required to address the specific needs for bone fracture treatment and to reduce complication rates. In the present study, we specified the measurement of local bone strength as a potential method of identifying patients at high risk of construct failure once threshold values have been defined. For such patients, new techniques or implant systems can be used to address the specific requirements of osteoporotic fracture fixation. Implant augmentation is such a technique that increases the bone–implant interface by cement injection. Despite the superiority of augmentation in biomechanical studies [7, 8, 11, 13, 19–21], no clinical study has shown a significant benefit following augmentation [22]. Hence, it is demonstrated that an objective tool to identify patients who would benefit from additional implant augmentation is required, especially taking into consideration the fact that the augmentation procedure is associated with risks and possible complications for the patient.

This study has some limitations inherent to all cadaveric studies. First, a small sample size was used, however, due to ethical reasons and limited availability of human osteoporotic bones, we aimed to keep the number of specimens as low as possible. Second, the determination of bone quality using DensiProbe^™^ was performed in a laboratory setting under ideal conditions with equipment unavailable for intraoperative application. Third, HR-pQCT data were used for BMD calculation, which is not the clinical standard. Fourth, an unconstrained setup with a proximal ball-and-socket joint and a distal attachment to the specimen via an individual mold with a seesaw was used for cyclic loading, however, it allowed for loading close to the physiologic conditions with generation of clinically relevant modes of failure.

The local bone quality at the distal femur indicates that in plated distal femur fractures, the distal posterior screws seem to be key and should be used. Measurement of the local bone strength with DensiProbe^™^ is one possibility to determine the risk of construct failure, therefore, thresholds need to be defined.
